# Detection of multiple mycetoma pathogens using fungal metabarcoding analysis of soil DNA in an endemic area of Sudan

**DOI:** 10.1371/journal.pntd.0010274

**Published:** 2022-03-11

**Authors:** Hiroki Hashizume, Suguru Taga, Masayuki K. Sakata, Mahmoud Hussein Mohamed Taha, Emmanuel Edwar Siddig, Toshifumi Minamoto, Ahmed Hassan Fahal, Satoshi Kaneko

**Affiliations:** 1 School of Tropical Medicine and Global Health, Nagasaki University, Nagasaki, Japan; 2 Department of Ecoepidemiology, Institute of Tropical Medicine (NEKKEN), Nagasaki University, Nagasaki, Japan; 3 Graduate School of Human Development and Environment, Kobe University, Kobe, Japan; 4 Mycetoma Research Center, University of Khartoum, Khartoum, Sudan; University of Maryland School of Medicine, UNITED STATES

## Abstract

Mycetoma is a tropical disease caused by several fungi and bacteria present in the soil. Fungal mycetoma and eumycetoma are especially challenging to treat; therefore, prevention, early diagnosis, and early treatment are important, but it is also necessary to understand the geographic distribution of these pathogenic fungi. In this study, we used DNA metabarcoding methodology to identify fungal species from soil samples. Soil sampling was implemented at seven villages in an endemic area of Sennar State in Sudan in 2019, and ten sampling sites were selected in each village according to land-use conditions. In total, 70 soil samples were collected from ground surfaces, and DNA in the soil was extracted with a combined method of alkaline DNA extraction and a commercial soil DNA extraction kit. The region for universal primers was selected to be the ribosomal internal transcribed spacer one region for metabarcoding. After the second PCR for DNA library preparation, the amplicon-based DNA analysis was performed using next-generation sequencing with two sets of universal primers. A total of twelve mycetoma-causative fungal species were identified, including the prime agent, *Madurella mycetomatis*, and additional pathogens, *Falciformispora senegalensis* and *Falciformispora tompkinsii*, in 53 soil samples. This study demonstrated that soil DNA metabarcoding can elucidate the presence of multiple mycetoma-causative fungi, which may contribute to accurate diagnosis for patient treatment and geographical mapping.

## Author summary

Mycetoma, a chronic subcutaneous and cutaneous disease, designated as a "neglected tropical disease," is prevalent in dry and hot climates. Fungal mycetoma is caused by more than 50 species of soil-dwelling pathogenic fungi, and its diagnosis and treatment can be challenging. The prevention of infection and early diagnosis and treatment are essential, and for this purpose, environmental assessment to understand the fungal habitat is necessary. In this study, we performed DNA metabarcoding analysis using next-generation sequencing (NGS) for mycetoma pathogens from environmental soil samples in Sudan. The results suggest that multiple causative agents of fungal mycetoma are widespread regardless of the environment and can be a source of infection anywhere in an endemic area. Based on the results of this study, we expect that the investigation of fungi in soil using NGS technology may help identify infection routes and create risk maps for the prevention of mycetoma.

## Introduction

Mycetoma is a chronic granulomatous and disabling inflammatory disease caused by specific groups of bacteria (actinomycetoma) or fungi (eumycetoma). It typically affects people living in poor, remote communities in tropical and subtropical regions within the so-called mycetoma belt, located between latitude 15°S and 30°N [1]. Most of the causative microorganisms inhabit the soil and invade the human body through minor unnoticed wounds on the skin, mainly in the foot and hand, and multiply to form multiple painless subcutaneous mass lesions that discharge seropurulent grains [1–4]. As the lesions progress, the microorganisms invade more deeply into tissues and bones, which can lead to amputation of the affected limb and is sometimes fatal [5]. The detailed epidemiological characteristics, such as the route of transmission, incubation period, prevalence, and incidence, have not been elucidated due to its chronic slow progression, with patients only visiting the hospital after being infected for years [6,7].

Between actinomycetoma and eumycetoma, the latter causes serious public health problems, because there is no effective short-term medicine compared to the former, which is treated by existing antibiotics. Therefore, prevention and screening programs are needed for early detection and treatment before the lesions reach vital parts of the body. It is thus necessary to understand the geographical risk distribution of the infective fungi. Because the causative fungal pathogens exist in the soil in endemic areas, it is possible to determine the distribution of infection by analyzing collected soil samples from areas in or near-endemic areas to confirm their presence. Before now, only soil sampling surveys have been conducted explicitly targeting only *Madurella mycetomatis*, the leading causative agent in Sudan [4]. However, to date, over 50 species of mycetoma-causative fungi have been reported around the world [8], and some patients are simultaneously infected with multiple fungi [9–13]. To understand the distributions of the causative fungi, it is necessary to comprehensively capture all fungi in an endemic area’s soil.

To comprehensively capture and analyze large quantities of DNA information simultaneously, metabarcoding using next-generation sequencing (NGS) is used as a high throughput approach in several scientific fields. This technique has been applied for soil microbiome research to detect environmental DNA (eDNA) from soil samples in the fields of soil microbial ecology, environmental science, and botany [14–16]. By applying this metabarcoding technology, it is possible to capture all the causative fungi in the soil for environmental surveillance of mycetoma, which will be critical for eumycetoma prevention measurements and control programs.

In this study, we performed a metabarcoding technology-based soil sampling survey in an endemic area to establish the geographical risk distribution of eumycetoma.

## Methods

### Study area and soil sampling

**Fig 1:**
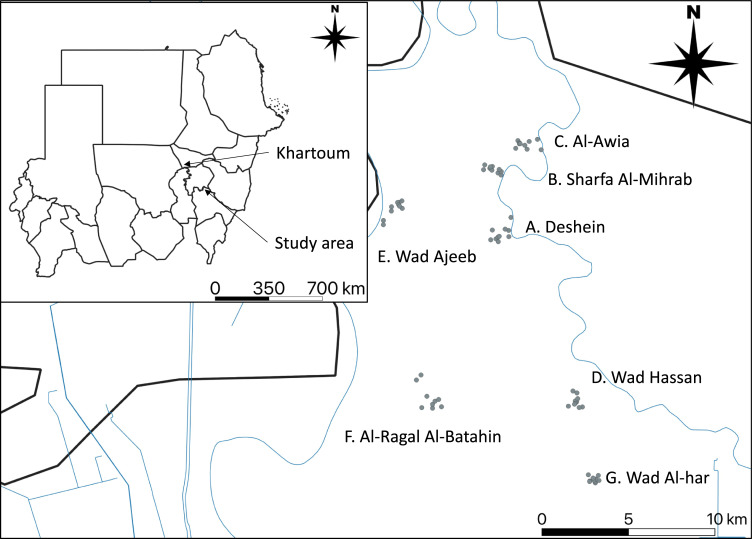
The geographical location of areas where soil samples were collected in this study. Each point on the map presents a sample collection site. Country and state maps were obtained from the GADM database under a CC BY license (https://geodata.ucdavis.edu/gadm/gadm4.0/shp/gadm40_SDN_shp.zip). Waterways (blue line) data were downloaded from the OpenStreetMap project (OpenStreetMap contributors) under a CC BY-SA 2.0 license (www.openstreetmap.org) through the platform Geofabrik (https://download.geofabrik.de/africa/sudan-latest-free.shp.zip). The map was created using the QGIS Geographic Information System, Open Source Geospatial Foundation Project, under a CC BY-SA 3.0 license (http://qgis.osgeo.org).

We chose ten villages in the state of Sennar, Sudan, located about 250 km south-east of Khartoum, where a mycetoma clinic is operated by the Mycetoma Research Centre (MRC), University of Khartoum, to manage mycetoma patients (Fig 1). Ten sampling sites where people gather or were near water sources were selected per village; we planned 100 soil sample collections from 10 villages. Each sampling site was categorized according to the land usage at the site as follows: 1) inside a cow fence, 2) dryland, 3) farmland, 4) riverside farm, and 5) road. The sampling survey was conducted for two days using a mobile data collection system, Open Data Kit (available online: https://opendatakit.org/) [17]. The soil sampling survey was conducted from October 16 to 17, 2019. The average temperature of Senner in October of 1991–2020 was 29.64°C (range: 22.28°C–37.05°C), and the average precipitation was 24.11 mm (sourced from the World Bank Climate Change Knowledge Portal) [18]. Soil samples were collected with 50 ml Falcon tubes from surfaces that none of the team members had stepped on using disposable plastic shovels [19]. The gloves and disposable shovels were changed at every location, and shoe covers were worn at every village to avoid contamination. The Falcon tubes were packed in a small plastic bag and stored in a styrofoam box with dry ice to minimize DNA degradation. Samples from the first day were kept in an empty −30°C freezer provided by the village head. After completing sampling on the second day, soil samples in the tubes were packed in styrofoam boxes with additional dry ice, transported to Khartoum by land, then stored in a −30°C freezer immediately upon arrival at MRC (see Supplemental method 1 in S1 Protocol for more details).

### DNA extraction

The DNA extraction protocol was broadly followed as previously reported to increase the amount of DNA extracted from the soil samples [19,20]. We used the integrated method of alkaline DNA extraction with ethanol precipitation and a commercial DNA extraction kit for soil samples (PowerSoil DNA Isolation Kit, Qiagen, Germany). For each sample, 9 g of soil was used for DNA extraction. During this process for each village, one negative control was obtained using 9 g of distilled water. The final DNA solutions were dissolved into the elusion buffer of the kit (see Supplemental method 2 in S1 Protocol for more details).

### Universal primers

**Fig 2:**
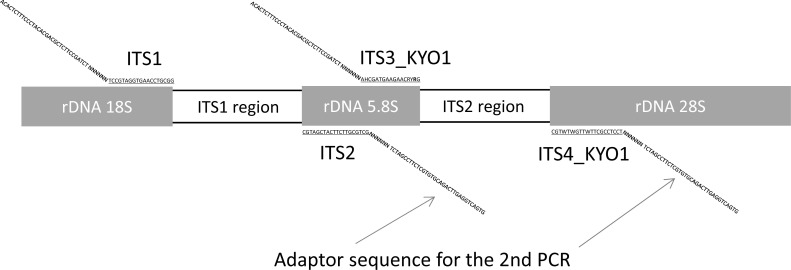
Map of ribosomal DNA genes with universal primers. The internal transcribed spacer 1 (ITS1) and 2 (ITS2) regions were targeted to identify causative species. The primer sets ITS1/ITS2 and ITS3_KYO1/ITS4_KYO1 were used as universal primers for the MiSeq system.

To choose universal primers for metabarcoding on the MiSeq platform (Illumina, USA), we cataloged mycetoma-causing fungi species from previous studies, which resulted in 29 genera with 55 species (including three at genus-level classification) (S1 Table). The internal transcribed spacer (ITS) regions and 18S ribosomal DNA are generally used for the identification of fungal species [21]. The accession of each causative fungus in the GenBank database by the National Center for Biotechnology Information (NCBI) was checked, and subsequently, the ITS1 and ITS2 regions were chosen as universal primer targets (S1 Table). Then, the sequences of 52 species available in GenBank were downloaded and subjected to multiple alignments using MUSCLE in the Unipro UGENE program (version 40.0) [22,23]. The set of aligned sequences was inspected visually with primers reported previously [24–26]. The universal primers that covered as many species as possible in the data set were adopted as our MiSeq primers. Primers sets named ITS1/ITS2 and ITS3_KYO1/ITS4_KYO1 shared sequences well with the target fungal genes [24,26]. Therefore, primers were designed for MiSeq in combination with ITS1/ITS2 and ITS3_KYO1/ITS4_KYO1, and six random hexamers (N) and adapter sequences (Fig 2 and Table 1).

**Table 1:** Primer sequences for the MiSeq analysis.

Primers	Sequence	Original primer names
ITS1_U	5’-ACACTCTTTCCCTACACGACGCTCTTCCGATCT NNNNNN TCCGTAGGTGAACCTGCGG-3’	ITS1^a^
ITS2_U	5’-GTGACTGGAGTTCAGACGTGTGCTCTTCCGATCT NNNNNN GCTGCGTTCTTCATCGATGC-3’	ITS2^a^
ITS3_KYO1_U	5’- ACACTCTTTCCCTACACGACGCTCTTCCGATCT NNNNNN AHCGATGAAGAACRY**R**G- 3’	ITS3_KYO1^b^
ITS4_KYO1_U	5’- GTGACTGGAGTTCAGACGTGTGCTCTTCCGATCT NNNNNN TCCTCCGCTTWTTGWTWTGC- 3’	ITS4_KYO1^b^

Underlined sequences are traditionally used as universal fungal primers. The bolded base was slightly modified from the original.

^a^ reference [24]

^b^ reference [26]

### PCR amplification

Targeting ribosomal ITS1 and ITS2, we employed two-step PCR library preparations. The first was to amplify specific regions in eumycetoma-causing species, and the second was for the attachment of distinguishable tags for metabarcoding, as reported previously [27,28]. PCR reagents and PCR products were prepared in separate rooms to avoid unanticipated DNA contamination. Three replicates were amplified for the first-round PCR (1st PCR) for each sample using the forward and reverse primers targeting the fungal ITS1 and ITS2 regions (Table 1). A total of 25 μl PCR reaction volume contained 0.5 U of KOD-Plus-Neo (Toyobo, Japan), 2.5 μl of 10× buffer for KOD-Plus-Neo (Toyobo), 2.5 μl of 2 mM dNTPs solution, 1.5 μl of 25 mM MgSO4 solution, 0.75 μl of 10 μM each primer, and 2 μl DNA extract. For the PCR targeting ribosomal ITS2, ten-fold dilutions of 17 colored DNA extracts were also used to reduce the influence of PCR inhibition (S2 Table). The PCR was carried out with 40 cycles at 94°C for 20 s, 65°C for 30 s (57°C for PCR targeting ribosomal ITS2), and 68°C for 30 s, then completed with a final 68°C for 5 min. Then, DNA purification was performed for the 1st PCR products with SPRIselect Reagent Kit (Beckman Coulter, USA) and quantified using a Qubit fluorometer 3.0 (Thermo Fisher Scientific, USA). Preliminary checks using gel electrophoresis were performed to confirm PCR products. The dilution factor was calculated for each sample to 0.1 ng/μl, and the average dilution rate was applied to the negative controls. Subsequently, all DNA samples from the soils were diluted for the second-round PCR (2nd PCR).

Next, the 2nd PCR, which added a unique 8-bp index and MiSeq adaptor sequences at each end of the amplicons, was performed for Illumina MiSeq. The PCR was carried out in a 12 μl reaction volume using 6 μl of 2× KAPA HiFi HotStart ReadyMix (KAPA Biosystems, USA), 2 μl of forward and reverse primers with index and adaptor sequences (1.8 μM), 1 μl of combined 1st PCR DNA templates, and 1 μl of ultrapure water under the following thermal cycler profile: 95°C for 3 min, followed by 12 cycles of 98°C for 20 s and 72°C for 30 s, finally 72°C for 5 min. All the 1st PCR products were combined into one and then applied to the MiSeq Reagent Kit v3 for 2 × 300 bp (600 cycles) (Illumina) with PhiX Control v3 (Illumina). Detailed protocols are described in the supplement (see Supplemental method 3 in S1 Protocol).

### Bioinformatics

According to previously reported methods, the raw MiSeq data were pretreated and analyzed using USEARCH v10.0.240 [19,29]. Paired-end reads were combined; meanwhile, reads with low quality, short length, and many differences (>5 positions) in the merged region were discharged. Next, primer sequences, low-quality reads, and short reads were removed. Dereplication was performed to the set of reads; then denoising was conducted to generate amplicon sequence variants (ASVs). Chimeric and minor (<10 reads) ASVs were removed.

Then the data were analyzed systematically and robustly, and species-level identification by ASVs was conducted through BLAST+ [30] searches with a 97% identity threshold and 90% query cover of the entire query sequence [27,31,32]. Since a small number of ASVs occurred in negative control samples, these ASV sequences were first removed from the sequence data of all samples. We downloaded and used the UNITE database v8.2 (2020-02-04) (https://unite.ut.ee/) [33,34]. After a homology search with BLASTN, the species names of the eumycetoma-causative fungi were extracted from the results. To ensure identification at the level of a single species, we checked the species names manually, whether best (i.e., single and top) or not, with reference to the value of identity and E-values (see Supplemental method 4 in S1 Protocol for more details).

## Statistical analysis

One-way analysis of variance (ANOVA) followed by the Turkey-Kramer test was conducted to test whether there were differences in the number of causative species by land use of the sampling sites, using R (version 4.1.2, 64-bit).

## Results

### Soil sampling and DNA extraction

**Fig 3:**
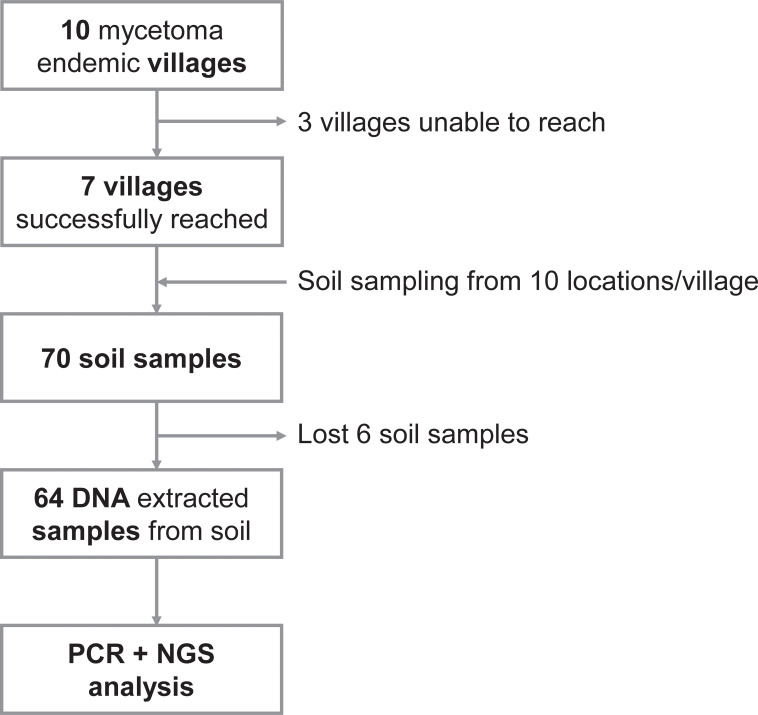
Schematic diagram of soil sampling prior to NGS analysis.

Finally, 70 soil samples were retrieved from seven villages (Al Awia, Al Ragal Al Batahin, Deshein, Sharfa Al Mihrab, Wad Ajeeb, Wad Al-Har, Wad Hassan) (Fig 3). We could not approach three villages due to flooding. Although the weather was sunny during the sampling, one village (Wad Al-Har) was muddy, except for the center of the village. Few riverside farm samples were collected from the targeted location due to secured blockades and fences; therefore, samples were collected from the closest location.

A total of 64 DNA samples with six negative controls were extracted, and 17 of the samples were colored with soil components. During the laboratory work process, seven samples, including one negative control, were lost due to a malfunction of the centrifuge separator (Fig 3).

### PCR amplification and MiSeq sequencing

After the 1st PCR targeting ribosomal ITS1 to amplify fungal DNA, preliminary checks found target size bands from 37 samples, but no band was found in the negative controls. Hence, for the MiSeq for ribosomal ITS2, we did only an initial check for some samples.

As an outcome of MiSeq for ribosomal ITS1, 3,158,851 pair-end sequences (reads) were obtained in total from the soil and negative control samples. The averages (±SD) of soil samples and negative controls (blanks of DNA extraction and the 1st PCR) were 24,631 (± 22,865) reads (lowest number 19 reads; highest number 161,247 reads) and 306 (± 346) reads, respectively. After processing with USEARCH, 7,357 ASVs were found in all DNA samples. The averages (±SD) of soil samples and negative controls/PCR blanks of the 1st PCR were 114 (± 86.0) reads and 6.1 (± 5.7) reads, respectively. For ribosomal ITS2, a total of 21,177,128 pair-end reads, an average of 130,569 (± 126,731) reads (lowest number 14 reads; highest number 679,229 reads) for soil samples and 1,248 (± 2,208) for negative controls, and a total of 10,780 ASVs, an average of 133 (± 107) reads for soil samples, and 5.8 (± 9.8) reads for negative controls, were obtained.

### Eumycetoma-causative fungi detected in the samples

In the BLAST results based on the UNITE database, nine genera and twelve species of causative fungi, including the primary pathogen in Sudan, *M*. *mycetomatis*, and *Falciformispora senegalensis* and *Falciformispora tompkinsii*, were retrieved (Table 2). The number of detected species was counted based on land use; there was a significant difference only between road (3.3 ± 1.5) and dryland (1.9 ± 1.0) (p = 0.045) (S1 Fig).

**Table 2:** Causative fungal species hit with 97% identity threshold and top hit species.

Village	Land use	*Aspergillus terreus*	*Amesia atrobrunnea*	*Curvularia lunata*	*Exserohilum rostratum*	*Falciformispora senegalensis*	*Falciformispora tompkinsii*	*Fusarium solani*	*Madurella fahalii*	*Madurella mycetomatis*	*Madurella tropicana*	*Medicopsis romeroi*	*Phaeoacremonium parasiticum*
A. Deshein	Farm 1	-	-	c	b	-	-	-	-	-	-	-	-
	Farm 2	-	-	c	a	-	-	-	-	b	-	-	-
	Road	-	-	b	b	-	-	b	-	b	-	-	-
	Cattle 1	-	-	a	-	-	-	-	-	-	-	-	-
	Cattle 2	-	b	c	-	-	-	b	-	-	-	-	-
	Dryland	-	-	c	-	-	-	-	-	b	-	-	-
	Riverfarm	-	-	a	-	-	-	b	-	-	-	-	-
B. Sharfa Al-Mihrab	Farm 1	a	-	-	-	-	-	-	-	-	-	-	-
	Road	-	-	a	-	-	-	-	-	b	b	-	-
	Dryland 1	a	-	c	b	-	-	-	-	-	-	-	-
	Riverfarm 1	-	-	a	-	a	-	-	-	-	-	-	-
	Riverfarm 2	-	-	a	-	a	-	-	-	-	-	-	-
	Dryland 2	-	-	-	b	-	-	-	-	-	-	-	-
	Farm 2	-	-	c	-	-	-	b	b	-	-	-	-
C. Al-Awia	Farm 1	-	-	c	-	-	-	-	b	-	-	-	-
	Farm 2	-	-	c	-	-	-	-	b	-	-	-	-
	Road 1	-	-	c	b	-	-	b	-	b	b	-	-
	Road 2	-	b	c	b	-	-	b	-	b	b	-	-
	Cattle	-	-	a	-	-	-	-	-	-	b	-	-
	Dryland 1	-	-	a	-	-	-	-	-	-	-	-	-
	Riverfarm 1	-	-	c	-	-	a	b	-	-	-	-	-
	Riverfarm 2	-	-	c	-	-	-	-	-	-	-	-	-
	Dryland 2	-	-	c	-	-	-	-	-	-	-	-	-
D. Wad Hassan	Farm 1	-	-	c	c	-	-	b	-	-	-	-	-
	Riverfarm 1	a	-	c	-	-	-	b	-	-	-	-	-
	Road 1	a	-	c	-	-	-	-	b	-	-	-	b
	Road 2	-	-	b	-	-	-	b	-	b	-	-	-
	Farm 2	-	b	c	-	-	-	b	-	b	-	-	-
	Dryland 1	a	-	c	-	b	-	-	-	-	-	b	-
	Dryland 2	a	-	c	-	-	-	-	-	-	-	-	-
	Riverfarm 2	-	-	c	-	-	-	-	b	-	-	-	-
E. Wad Ajeeb	Farm 1	a	-	c	-	-	-	-	-	b	-	-	-
	Farm 2	-	-	c	b	-	-	-	-	b	-	-	-
	Road 1	-	-	-	b	-	-	-	b	-	-	-	-
	Road 2	-	-	b	-	-	-	-	-	-	-	-	-
	Dryland 1	-	-	a	-	-	-	-	b	b	-	-	-
	Dryland 1	a	-	-	-	-	-	-	-	-	-	-	-
	Riverfarm 1	-	-	c	-	-	-	b	b	-	-	-	-
	Riverfarm 2	-	-	c	-	-	-	-	-	-	-	c	-
F. Al-Ragal Al-Batahin	Dryland	-	-	c	-	-	-	-	-	-	-	-	-
	Farm 1	a	-	c	-	-	-	-	-	-	-	-	-
	Farm 2	a	-	c	b	-	-	-	-	-	-	-	-
	Cattle	-	-	a	-	-	-	-	-	-	-	-	-
	Road	-	-	b	b	-	-	b	-	b	-	b	-
	Farm 1	-	-	c	-	-	-	b	b	-	-	-	-
	Farm 2	-	-	c	-	-	-	-	-	-	-	-	-
G. Wad Al-har	Road 1	-	-	c	-	-	-	-	b	b	-	-	-
	Farm 1	-	-	c	-	-	-	b	-	b	-	-	-
	Farm 2	-	-	c	-	-	-	-	-	-	-	-	-
	Road 2	-	-	c	-	-	-	-	-	-	-	-	-
	Road 3	-	-	a	-	-	c	-	-	-	-	-	-
	Farm 2	-	-	a	-	-	-	-	-	b	-	-	-
	Farm 3	-	b	c	-	-	-	b	-	-	-	-	-

Letters indicate the following: a, detected species the MiSeq targeting the ribosomal ITS1 region; b, ribosomal ITS2; c, species hit in both analyses. Only locations where the target fungi were detected are shown.

## Discussion

In this study, we detected several eumycetoma-causing fungi simultaneously from soils of different land uses in an endemic area using a metabarcoding technology-based soil sampling survey method. Our analysis identified twelve species of eumycetoma pathogens from soil DNA samples, including the principal causative agent, *M*. *mycetomatis*. Only two previous studies detected the DNA of *M*. *mycetomatis* and other causative species from the soil or *Acacia* thorns and other environmental agents [4,16]. However, the simultaneous detection of multiple pathogenic fungi is scientifically warranted because 1) over 50 species of mycetoma-causative fungi are reported worldwide [8], 2) mycetoma is caused by various fungi classes [5,35], 3) multiple fungi are found even in a single lesion of a patient [36], and 4) it has been reported that fungi collected from patient lesions and cultured are present in the soil [6,7,35].

As previous reports have mentioned, mycetoma is distributed widely within arid areas, called the mycetoma belt [1]. In this study, the field sites were selected from the state of Sennar, which is in a dry climate. Soil samples from several different land-use conditions in the endemic region of eumycetoma were also collected and analyzed. As a result, surprisingly, the DNA of the twelve species known as causative agents, including the most important, *M*. *mycetomatis*, and some other rare pathogenic species (i.e., *Aspergillus* and *Curvularia*), were detected from 83% (53/64) of the soil samples. In regard to specificity and sensitivity, this study shows the advantage of multiple detections of mycetoma-causative pathogens. Our results showed that regardless of the environmental conditions, various eumycetoma pathogens are ubiquitous in the soils of these areas. In addition, the analysis showed a statistical difference between roads and drylands, which indicates that some environmental agents may affect the existence of pathogens in soil, though this requires further study. More extensive field sampling, including soil composition or climate data, may uncover the environmental preferences of mycetoma-causative fungi. Moreover, the outcome of soil fungal metabarcoding can be used to visualize a risk map of the causative agents with remote sensing data, as with previous studies using clinical data [37,38]. This can lead to robust estimates of the environmental risk factors associated with mycetoma, which have not been elucidated over its long history.

Furthermore, our findings can help with diagnosis or preventive measures for patients. Clarifying the diversity of fungi, including eumycetoma agents, in patients’ villages might contribute to the accurate diagnosis of the pathogens. Our data also suggest that protective measures such as wearing shoes should be strongly promoted for people in mycetoma-endemic areas.

Based on the data obtained in this study, we constructed a system of amplicon sequencing and metabarcoding techniques targeting a group of mycetoma-causative organisms. Considering the PCR conditions, we faced a major limitation: some extracted DNA solutions were brown-colored (S2 Table), which means that the samples might have been contaminated with PCR inhibitors such as humic acids in the soil. Here, we applied a DNA extraction method developed for sedimentary soils of river bottoms [20]; however, the soil moisture contents were different for each land use, which might have influenced the PCR amplification. For subsequent MiSeq analysis targeting ribosomal ITS2, the colored DNA samples were diluted in the 1st PCR to produce accurate results. Accordingly, most target DNA was successfully amplified (i.e., reads and ASVs) compared with the same samples that were not diluted.

In this study, we focused on species-level detection of causative agents of eumycetoma. Therefore, the number of sequences that could not be identified as species might be underestimated, which means there were possibly more pathogens in the sampling sites. Thus, further identifying species from mycetoma specimens and registration in gene databases is still needed. In our results, *Aspergillus terreus* was only identified at the species level for the ribosomal DNA ITS1 region, although the *Madurella* genus was identified in the analysis for ITS2. For pathogenic species identification, at this point, using both the ITS1 and ITS2 regions would lead to more accurate results.

## Conclusion

The metabarcoding technology-based soil sampling survey method can detect multiple causative fungi from soil samples in an endemic area, including the major pathogen, *M*. *mycetoma*. Applying the technology to construct a geographic distribution of causative fungi provides essential and fundamental information for preventive measures, diagnosis considering regional characteristics and fungi distribution, and the development of therapeutic agents against mycetoma.

## Supporting information

S1 Table Causative microorganisms of eumycetoma in the world. We used sequences of the accession numbers below to choose universal primers.
(XLSX)

S2 Table Colored DNA samples after extraction. These samples had inadequately remove soil-derived PCR inhibitors. Each letter shows a sampling village: A. Deshein, B. Sharfa Al-Mihrab, C. Al-Awia, D. Wad Hassan, E. Wad Ajeeb, F. Al-Ragal Al-Batahin, and G. Wad Al-har. NCs, negative controls at DNA extraction.
(XLSX)

S1 Fig Number of detected eumycetoma-causative species per land-use category. (EPS)

S1 Protocol Supplemental methods. Supporting information of methods. Supplemental method 1. Soil sampling. Supplemental method 2. DNA extraction. Supplemental method 3. PCR amplification. Supplemental method 4. Bioinformatics.
(DOCX)

## References

[1] Zijlstra EE, van de Sande WWJ, Welsh O, Mahgoub ES, Goodfellow M, Fahal AH. Mycetoma: a unique neglected tropical disease. The Lancet Infectious Diseases. 2016;16: 100–112. doi: 10.1016/S1473-3099(15)00359-X 26738840

[2] Omer RF, Seif EL Din N, Abdel Rahim FA, Fahal AH. Hand Mycetoma: The Mycetoma Research Centre Experience and Literature Review. PLoS Neglected Tropical Diseases. 2016;10: 1–13. doi: 10.1371/journal.pntd.0004886 27483367 PMC4970814

[3] Fahal AH. Mycetoma: A thorn in the flesh. Transactions of the Royal Society of Tropical Medicine and Hygiene. Royal Society of Tropical Medicine and Hygiene; 2004. pp. 3–11. doi: 10.1016/S0035-9203(03)00009-9 14702833

[4] Ahmed A, Adelmann D, Fahal A, Verbrugh H, Van Belkum A, De Hoog S. Environmental occurrence of Madurella mycetomatis, the major agent of human eumycetoma in Sudan. Journal of Clinical Microbiology. 2002;40: 1031–1036. doi: 10.1128/JCM.40.3.1031-1036.2002 11880433 PMC120253

[5] Fahal A, Mahgoub ES, EL Hassan AM, Abdel-Rahman ME, Alshambaty Y, Hashim A, et al. A New Model for Management of Mycetoma in the Sudan. PLoS Neglected Tropical Diseases. 2014;8. doi: 10.1371/journal.pntd.0003271 25356640 PMC4214669

[6] van de Sande WWJ. Global Burden of Human Mycetoma: A Systematic Review and Meta-analysis. Vinetz JM, editor. PLoS Neglected Tropical Diseases. 2013;7: e2550. doi: 10.1371/journal.pntd.0002550 24244780 PMC3820768

[7] Fahal AH. Mycetoma: A global medical and socio-economic dilemma. PLoS Neglected Tropical Diseases. 2017;11. doi: 10.1371/journal.pntd.0005509 28426654 PMC5398501

[8] Mhmoud NA, Santona A, Fiamma M, Siddig EE, Deligios M, Bakhiet SM, et al. Chaetomium atrobrunneum causing human eumycetoma: The first report. Xue C, editor. PLOS Neglected Tropical Diseases. 2019;13: e0007276. doi: 10.1371/journal.pntd.0007276 31145740 PMC6542518

[9] Pilsczek FH, Augenbraun M. Mycetoma fungal infection: Multiple organisms as colonizers or pathogens? Revista da Sociedade Brasileira de Medicina Tropical. 2007;40: 463–465. doi: 10.1590/s0037-86822007000400017 17876471

[10] Mhmoud NA, Fahal AH, Mahgoub ES, van de Sande WWJ. The Combination of Amoxicillin-Clavulanic Acid and Ketoconazole in the Treatment of Madurella mycetomatis Eumycetoma and Staphylococcus aureus Co-infection. PLoS Neglected Tropical Diseases. 2014;8: 1–5. doi: 10.1371/journal.pntd.0002959 24945499 PMC4063734

[11] Kim JS. Case report: Case report. Canadian Family Physician. 2001;47: 788–789. 11340760 PMC2018417

[12] Bakshi R, Mathur DR. Incidence and changing pattern of mycetoma in western Rajasthan. Indian journal of pathology & microbiology. 2008;51: 154–155. doi: 10.4103/0377-4929.40433 18417892

[13] Bassiri-Jahromi S. Mycetoma in iran: causative agents and geographic distribution. Indian journal of dermatology. 2014;59: 529. doi: 10.4103/0019-5154.139889 25284877 PMC4171940

[14] Toju H, Sato H, Yamamoto S, Kadowaki K, Tanabe AS, Yazawa S, et al. How are plant and fungal communities linked to each other in belowground ecosystems? A massively parallel pyrosequencing analysis of the association specificity of root-associated fungi and their host plants. Ecology and Evolution. 2013;3: 3112–3124. doi: 10.1002/ece3.706 24101998 PMC3790555

[15] Matsuoka S, Kawaguchi E, Osono T. Temporal distance decay of similarity of ectomycorrhizal fungal community composition in a subtropical evergreen forest in Japan. FEMS Microbiology Ecology. 2016;92: 1–11. doi: 10.1093/femsec/fiw061 26989126

[16] Moussa TAA, Al-Zahrani HS, Almaghrabi OA, Abdelmoneim TS, Fuller MP. Comparative metagenomics approaches to characterize the soil fungal communities of western coastal region, Saudi Arabia. PLoS ONE. 2017;12: 1–13. doi: 10.1371/journal.pone.0185096 28934322 PMC5608318

[17] Hartung C, Anokwa Y, Brunette W, Lerer A, Tseng C, Borriello G. Open data kit: Tools to build information services for developing regions. ACM International Conference Proceeding Series. 2010. doi: 10.1145/2369220.2369236

[18] Sudan—Climatology | Climate Change Knowledge Portal. [cited 21 Jan 2022]. In: Climate Change Knowledge Portal [Internet]. Available from: https://climateknowledgeportal.worldbank.org/country/sudan/climate-data-historical

[19] Sakata MK, Yamamoto S, Gotoh RO, Miya M, Yamanaka H, Minamoto T. Sedimentary eDNA provides different information on timescale and fish species composition compared with aqueous eDNA. Environmental DNA. 2020;2: 505–518. doi: 10.1002/edn3.75

[20] Sakata MK, Watanabe T, Maki N, Ikeda K, Kosuge T, Okada H, et al. Determining an effective sampling method for eDNA metabarcoding: a case study for fish biodiversity monitoring in a small, natural river. Limnology. 2021;22: 221–235. doi: 10.1007/s10201-020-00645-9

[21] Schoch CL, Seifert KA, Huhndorf S, Robert V, Spouge JL, Levesque CA, et al. Nuclear ribosomal internal transcribed spacer (ITS) region as a universal DNA barcode marker for Fungi. Proceedings of the National Academy of Sciences of the United States of America. 2012;109: 6241–6246. doi: 10.1073/pnas.1117018109 22454494 PMC3341068

[22] Edgar RC. MUSCLE: A multiple sequence alignment method with reduced time and space complexity. BMC Bioinformatics. 2004;5: 1–19. doi: 10.1186/1471-2105-5-1 15318951 PMC517706

[23] Okonechnikov K, Golosova O, Fursov M, Varlamov A, Vaskin Y, Efremov I, et al. Unipro UGENE: a unified bioinformatics toolkit. Bioinformatics. 2012;28: 1166–1167. doi: 10.1093/bioinformatics/bts091 22368248

[24] White TJ, Bruns T, Lee S, Taylor J. Amplification and Direct Sequencing of Fungal Ribosomal Rna Genes for Phylogenetics. PCR Protocols. 1990; 315–322. doi: 10.1016/b978-0-12-372180-8.50042–1

[25] Usyk M, Zolnik CP, Patel H, Levi MH, Burk RD. Novel ITS1 Fungal Primers for Characterization of the Mycobiome. mSphere. 2017;2: 1–11. doi: 10.1128/mSphere.00488-17 29242834 PMC5729218

[26] Toju H, Tanabe AS, Yamamoto S, Sato H. High-coverage ITS primers for the DNA-based identification of ascomycetes and basidiomycetes in environmental samples. PLoS ONE. 2012;7. doi: 10.1371/journal.pone.0040863 22808280 PMC3395698

[27] Miya M, Sato Y, Fukunaga T, Sado T, Poulsen JY, Sato K, et al. MiFish, a set of universal PCR primers for metabarcoding environmental DNA from fishes: Detection of more than 230 subtropical marine species. Royal Society Open Science. 2015;2. doi: 10.1098/rsos.150088 26587265 PMC4632578

[28] Sato Y, Mizuyama M, Sato M, Minamoto T, Kimura R, Toma C. Environmental DNA metabarcoding to detect pathogenic Leptospira and associated organisms in leptospirosis-endemic areas of Japan. Scientific Reports. 2019;9: 1–11. doi: 10.1038/s41598-018-37186-2 31024059 PMC6484013

[29] Edgar RC. Search and clustering orders of magnitude faster than BLAST. BIOINFORMATICS APPLICATIONS NOTE. 2010;26: 2460–2461. doi: 10.1093/bioinformatics/btq461 20709691

[30] Camacho C, Coulouris G, Avagyan V, Ma N, Papadopoulos J, Bealer K, et al. BLAST+: Architecture and applications. BMC Bioinformatics. 2009;10. doi: 10.1186/1471-2105-10-421 20003500 PMC2803857

[31] Fouts DE, Szpakowski S, Purushe J, Torralba M, Waterman RC, MacNeil MD, et al. Next Generation Sequencing to Define Prokaryotic and Fungal Diversity in the Bovine Rumen. PLoS ONE. 2012;7: 48289. doi: 10.1371/journal.pone.0048289 23144861 PMC3492333

[32] Vasar M, Andreson R, Davison J, Jairus T, Moora M, Remm M, et al. Increased sequencing depth does not increase captured diversity of arbuscular mycorrhizal fungi. Mycorrhiza. 2017;27: 761–773. doi: 10.1007/s00572-017-0791-y 28730541

[33] Abarenkov K, Zirk A, Piirmann T, Pöhönen R, Ivanov F, Nilsson RH, et al. UNITE general FASTA release for Fungi. UNITE Community. 2020. Available: https://plutof.ut.ee/#/doi/10.15156/BIO/786368

[34] Abarenkov K, Nilsson RH, Larsson KH, Alexander IJ, Eberhardt U, Erland S, et al. The UNITE database for molecular identification of fungi–recent updates and future perspectives. New Phytologist. 2010;186: 281–285. doi: 10.1111/j.1469-8137.2009.03160.x 20409185

[35] de Hoog GS, Ahmed SA, Najafzadeh MJ, Sutton DA, Keisari MS, Fahal AH, et al. Phylogenetic Findings Suggest Possible New Habitat and Routes of Infection of Human Eumyctoma. Wanke B, editor. PLoS Neglected Tropical Diseases. 2013;7: e2229. doi: 10.1371/journal.pntd.0002229 23696914 PMC3656121

[36] Frickmann H, Künne C, Hagen RM, Podbielski A, Normann J, Poppert S, et al. Next-generation sequencing for hypothesis-free genomic detection of invasive tropical infections in poly-microbially contaminated, formalin-fixed, paraffin-embedded tissue samples—A proof-of-principle assessment. BMC Microbiology. 2019;19: 1–19. doi: 10.1186/s12866-018-1372-8 30961537 PMC6454699

[37] Samy AM, van de Sande WWJ, Fahal AH, Peterson AT. Mapping the Potential Risk of Mycetoma Infection in Sudan and South Sudan Using Ecological Niche Modeling. PLoS Neglected Tropical Diseases. 2014;8: e3250. doi: 10.1371/journal.pntd.0003250 25330098 PMC4199553

[38] Hassan R, Simpson H, Cano J, Bakhiet S, Ganawa E, Argaw D, et al. Modelling the spatial distribution of mycetoma in Sudan. Transactions of The Royal Society of Tropical Medicine and Hygiene. 2021;115: 1144–1152. doi: 10.1093/trstmh/trab076 34037803 PMC8486737

