# Supplementary material for: Detection of multiple mycetoma pathogens using fungal metabarcoding analysis of soil DNA in an endemic area of Sudan
Source: PLoS Negl Trop Dis. 2022 Mar 11;16(3):e0010274. doi: 10.1371/journal.pntd.0010274 (PMC8942264; doi:10.1371/journal.pntd.0010274)
Supplement: S1 Protocol — Supporting information of methods. Supplemental method 1. Soil sampling. Supplemental method 2. DNA extraction. Supplemental method 3. PCR amplification. Supplemental method 4. Bioinformatics. (DOCX) [file pntd.0010274.s004.docx]

**S1 Protocol. Supplemental methods**

**Supplemental method 1. Soil sampling**

Ten villages were chosen as candidates (Abu Laila, Al Awia, Al Ragal Al Batahin, Amoud, Deshein, Om Sablango, Sharfa Al Mihrab, Wad Ajeeb, Wad Al-Har, and Wad Hassan). Google map satellite images were used to select ten locations per village to take soil samples, such as where people might gather or near water sources. Each site was categorized as having the following land usage: cattle (circular dark color land with thorny plants or a fence), dry land (light-colored land that is not a farm), farm (area with crops or plowed), river farm (farm near the river or water source), and road (road between houses). Coordinates of 100 locations were stored in a GPS receiver (Garmin GPSMAP 64st, USA), and these locations were reached with a pickup truck and on foot. Field sampling was performed for two days driving around the study area. At each site, location information was collected, and we took pictures using the Open Data Kit (ODK; available at https://opendatakit.org/) on an android device.

**Supplemental method 2. DNA extraction**

First, rocks were removed, and 9 g of soil sample was measured and powdered by shaking. A new autoclaved spatula was used for each sample. Six milliliters of 0.33 M NaOH solution, 3 ml of Tris-EDTA (TE) buffer (pH 6.7), and 500 µl of G2 DNA/RNA enhancer (Ampliqon A/S, Denmark) were added to 9 g of sediment samples in 50 ml centrifuge tubes, then vortexed evenly. Solutions were added on a different lab bench from the soil measuring table. After incubation at 94°C for 50 min, samples were centrifuged at 3,600 × *g* for 2 min at room temperature (RT). Then, 7.5 ml of resulting supernatants were extracted to new 50 ml tubes and neutralized by adding 7.5 ml of 1 M Tris-HCl (pH 6.7). Then, 1.5 ml of NaAc solution (pH 5.2) and 30 ml of 99.5% EtOH were added to the tube and incubated in a freezer (−30°C) for over 1 h. During this process for each village, 9 g of distilled water was included as a negative control. Next, the samples were separated by centrifugation at 3,600 × *g* for 40 min at RT, and then supernatants were decanted and discarded. The precipitates were transferred to a Power Bead Tube (PowerSoil DNA Isolation Kit, Qiagen, Germany) with a spatula, and the remaining pellets were retrieved by pipetting with 100 µl of purified water (for injection) to the bead tube. From here, DNA extraction followed the protocol of the PowerSoil DNA Isolation Kit (Protocol: Experienced User 3 to 22).

**Supplemental method 3. PCR amplification**

For the first-round PCR (1st PCR), a total 25 µl PCR reaction volume contained 0.5 U of KOD-Plus-Neo (Toyobo, Japan), 2.5 µl of 10× buffer for KOD-Plus-Neo (Toyobo), 2.5 µl of 2 mM dNTPs solution, 1.5 µl of 25 mM MgSO4 solution, 0.75 µl of 10 µM each primer, 2 µl DNA extract, and 14.5 µl ultrapure water. Thermal conditions for PCR were 95°C for 3 min, followed by 40 cycles of 94°C for 20 s, 65°C for 30 s (57°C for PCR targeting ribosomal ITS2), and 68°C for 30 s, and completed with a final 68°C for 5 min. The annealing temperature was determined by observing the bands by gel electrophoresis at several temperatures with a preliminary experiment. Three technical replicates were amplified for each sample. The same volume of ultrapure water was used as a negative control. The operation of the 1st PCR was divided four times and conducted once a day to diminish contamination risk.

DNA purification was performed for the 1st PCR products with a SPRIselect Reagent Kit (Beckman Coulter, USA) following the standard protocol after three replicates were mixed. The volume of SPRIselect added to samples was equal to each 1st PCR product (75 µl). Subsequently, the DNA concentration of each sample was quantified by a Qubit fluorometer 3.0 (Thermo Fisher Scientific, USA). For the analysis targeting ribosomal ITS1, the DNA content of soil samples ranged from 0.11 to 41.8 ng/µl and averaged 9.64 ng/µl. The average of negative controls for the DNA extraction and the PCR blanks were 0.13 and 0.18 ng/µl, respectively. For ribosomal ITS2, the DNA content ranged from 0.021 to 42.0 ng/µl and averaged 8.23 ng/µl. All DNA amplicons of negative controls and PCR blanks were of low concentration and out of the measurement range.

Then, all DNA samples were diluted with ultrapure water to 0.1 ng/µl. To perform an equivalent operation, DNA samples of negative controls for the DNA extraction and the PCR blanks for the MiSeq analyses of ribosomal ITS1 and ITS2 were diluted 96-fold (i.e., 0.1 ng/µl divided by the average of 9.64 ng/µl) and 89-fold with ultrapure water, respectively.

The second-round PCR (2nd PCR), which added a unique 8-bp index and MiSeq adaptor sequences at each end of the amplicons, was performed for Illumina MiSeq. The PCR was carried out in 12 µl reaction volumes using 6 µl of 2× KAPA HiFi HotStart ReadyMix (KAPA Biosystems, USA), 2 µl of forward and reverse primers with index and adaptor sequences (1.8 µM), 1 µl of combined 1st PCR DNA templates and 1 µl of ultrapure water, under the following thermal cycler profile: 95°C for 3 min, followed by 12 cycles of 98°C for 20 s and 72°C for 30 s, and finally 72°C for 5 min.

All the 2nd PCR products ware pooled in equal volumes. We checked the length of amplicons by an E-Gel Precast Agarose Electrophoresis System (Thermo Fisher Scientific, USA) and confirmed that DNA was in targeted 350-450 bp with an Agilent 2100 Bioanalyzer (Agilent Technologies, USA). The pooled DNA samples were adjusted to 4 nM and 1 nM for targeting ribosomal ITS1 and ITS2, respectively, to which 20% of the total was added DNA of PhiX Control v3 (Illumina, USA) for improving data quality. Eventually, the DNA library was applied to a MiSeq Reagent Kit v3 for 2 × 300 bp (600 cycles) (Illumina).

**Supplemental method 4. Bioinformatics**

Data of the MiSeq analysis were demultiplexed using MiSeq Reporter Software 2.6.2 (Illumina). Then, data preprocessing was performed using USEARCH v10.0.240. Paired-end reads were combined with the command "fastq_mergepairs" by default. Meanwhile, reads with a low-quality score (under a cutoff threshold of Phred score of 2), short length (<100 bp) after tail trimming, and many differences (> 5 positions) in the aligned region were discharged in this process. The commands of "fastx_truncate" and "fastq_filter" were used to remove primer sequences, low-quality reads with a rate of the expected error of > 1%, and reads less than 100 bp. Dereplication was performed to the set of reads using the command "fastx_uniques," then denoising was conducted with the command "unoise3" to generate amplicon sequence variants (ASVs). Chimeric and minor (< 10 reads) ASVs were removed.

Species identification of ASVs was conducted through BLAST searches for 97% identity with >90% query cover of the entire query sequence. At first, BLAST+ was installed in a local software environment. A small number of ASVs were found in negative control samples (B-NC, C-NC, E-NC, F-NC, G-NC, and DW4; the averages (±SD) were 9.7±10.3), which means possible contaminations occurred throughout the process of DNA library preparation. Therefore, these ASV sequences were removed from the sequence data of all samples. For the database, the UNITE database v8.2 (2020-02-04, includes global and 97% singletons) (<https://plutof.ut.ee/#/doi/10.15156/BIO/786369>), which is the reference database of a variety of fungi, was downloaded. After a homology search on the database with BLASTN with the default settings, we conducted word retrievals of 52 causative species names (using binomials including synonyms) on the BLAST results to find sites where their DNA (ASVs) was detected. Then, we manually checked whether the detected species were single top hits or not, with reference to the value of identity and E-values.
