# Supplementary material for: Detection of multiple mycetoma pathogens using fungal metabarcoding analysis of soil DNA in an endemic area of Sudan
Source: PLoS Negl Trop Dis. 2022 Mar 11;16(3):e0010274. doi: 10.1371/journal.pntd.0010274 (PMC8942264; doi:10.1371/journal.pntd.0010274)
Supplement: Attachment — Submitted filename: Response to Reviewers Comments_hh3.docx [file pntd.0010274.s005.docx]

Response to Reviewers Comments

We appreciate the valuable comments to the three reviewers. We have addressed all the suggestions raised by the reviewers and revised the manuscript accordingly.

To respond to the major comments, we added data on a second NGS analysis targeting the ribosomal ITS2 region, which we performed and analyzed after the original submission. We believe the combined results of the first and second NGS provided clear responses to the comments.

**Methods**

Reviewer #1: The methods used are the regular methods used in that study and there is no new and contribution to the methods used

**Response 1:**

We thank the kind comment. We have reconsidered the novelty of our research, which was the first environmental study to identify several eumycetoma-causing fungi simultaneously from soils with different land uses using NGS technology. However, concerning the novelty, the methodology itself was conventional. Therefore, we remove “a novel method” from the draft title.

Reviewer #2: Firstly, a thorough review of English is necessary.

**Response 2:**

We thank the suggestion. A professional editorial service has checked the revised draft.

The main claims of the paper is that eumycetoma is highly frequent in Sudán and the main etiologicla agent is M. mycetomatis. These claims are very significant to meet the proposed objective.

The authors have enough fairly the literature.

In general the manuscript is well organized, but not written clearly enough to be accessible to non-specialists.

**Response 3:**

The detailed experimental methods for PCR were added in Lines 169-179 and Lines 187-192.

The objective of the study is clearly established but not articulated with a clear testable hypothesis.

-The study design could be supplemented with culture to address the stated objectives.

**Response 4:**

Some fungal pathogens, including *Madurella mycetomatis*, can be cultured; however, in the revised draft, we detected twelve species. Cultivating this number of fungi requires much time, labor, and appropriate condition; therefore, we omitted this process in the current study. We thank this suggestion and hopefully will conduct cultivation or real-time PCR as another identification method in further research.

-The population is clearly described and appropriate.

-The sample size sufficient to ensure adequate power to address the study.

-The statistical analysis used were correct to support conclusions.

**Response 5:**

We appreciate these supportive comments.

-Specifically, in the sampled sites (Sennar State Suda), what are the most frequent eumycetoma agents reported? If the answer is M. mycetomatis, something is not well in the methodology. The authors tried to recover M. mycetomatis in culture?

**Response 6:**

*M. mycetomatis* is the most frequent agent of eumycetoma in this area, and in previous studies, cultivation of this species was conducted. In this revision, we performed another NGS analysis targeting the ribosomal ITS2 region and successfully detected *M. mycetomatis*.

-Some of additional work would improve the paper. For exemple, cultivation of different soils to get eumycetoma agents and phenotipically identify these ones. This methodology is laborious but not difficult.

**Response 7:**

We understand that species identification by culture is crucial. However, carrying out wide-area surveys and establishing a cultivation technique for a diverse fungal species is challenging. Still, it may be possible to culture only the main species, including *M. mycetomatis*. For further research, we believe that detecting fungi by culture, not only by eDNA, is essential for the verification of infectivity.

-The NGS is a technique that makes outstanding this paper, but unfortunately is not well explained and results no well exploited. Authors do not say if NGS was make at least by duplicate.

**Response 8:**

According to this comment, we performed replication of the NGS process (three replicates were amplified for the first-round PCR for each sample); also, we performed another NGS analysis targeting the ribosomal ITS2 in the same manner.

-Details of the methodology are no sufficient to allow the experiments to be reproduced.

**Response 9:**

A detailed methodology was added for PCR amplification.

-The analysis presented does not match the analysis plan. Madurella mycetomatis DNA was not detected by NGS after DNA extraction from soil.

-Results are clearly and completely presented, but are not that expected.

**Response 10:**

We achieved the initial plan with the second NGS analysis.

-The figures (Tables, Images) are of sufficient quality for clarity.

**Response 11:**

We appreciate the kind comment.

-I think authors should have done some previous experiments to standardize amplification conditions and then apply them to the NGS technique.

**Response 12:**

We agreed with this suggestion. We believe that improvements in the second NGS analysis, including dilution of DNA templates, have satisfied this recommendation.

Reviewer #3: -Are the objectives of the study clearly articulated with a clear testable hypothesis stated?

Yes

-Is the study design appropriate to address the stated objectives?

Yes

-Is the population clearly described and appropriate for the hypothesis being tested?

Yes

-Is the sample size sufficient to ensure adequate power to address the hypothesis being tested?

Yes

-Were correct statistical analysis used to support conclusions?

Yes

-Are there concerns about ethical or regulatory requirements being met?

No

**Response 13:**

We appreciate the kind evaluations.

**Results**

Reviewer #1: ok

**Response 14:**

We appreciate the kind evaluation.

Reviewer #2: -The analysis presented does not match the analysis plan. Madurella mycetomatis DNA was not detected by NGS after DNA extraction from soil.

-Results are clearly and completely presented, but are not that expected.

**Response 15:**

We achieved the initial plan with the second NGS analysis.

-The figures (Tables, Images) are of sufficient quality for clarity.

**Response 16:**

We appreciate the kind comment.

Reviewer #3: -Does the analysis presented match the analysis plan?

Yes

-Are the results clearly and completely presented?

Yes

-Are the figures (Tables, Images) of sufficient quality for clarity?

Yes; please make sure the map employed complies with the journal copyright requirements.

**Response 17:**

We added the following sentence in the description of Fig 1.

“Country and state maps were obtained from the GADM (https://gadm.org/index.html), and waterways (blue line) data were downloaded from the OpenStreetMap project (https://www.openstreetmap.org). The map was created using the QGIS Geographic Information System, Open Source Geospatial Foundation Project (http://qgis.osgeo.org).”

**Conclusions**

Reviewer #1: there is no conclusion on the study but it is general sentences not supported to the results obtained

**Response 18:**

We thank the crucial comment. After adding the data of the second NGS analysis, we edited the conclusion in Lines 335-336, with the sentence as below. We hope that the conclusion supports the obtained results.

“The metabarcoding technology-based soil sampling survey method can detect multiple causative fungi from soil samples in an endemic area, including the major pathogen, *M. mycetoma*.”

Reviewer #2: Discussion

In Discussion, authors say that that they did not find M. mycetomatis because of some PCR inhibitors. Why was this phenomenon not present with the other fungal agents?

**Response 19:**

We appreciate the scientific comment for discussion. Based on the second NGS analysis results, we modified the Discussion. PCR inhibitors may affect the fungal DNA detection of the 1st PCR step. However, we believe that the identification of *M. mycetomatis* was determined by the target gene region or primer sequences.

-The conclusions are not supported by the data presented, but only by the method advantage.

**Response 20:**

We thank the comment. We think the data meets our conclusion in this revised draft with the second NGS analysis.

-The limitations of analysis considered by authors are described.

-The public health relevance is addressed.

**Response 21:**

We appreciate the kind evaluation.

Reviewer #3: -Are the conclusions supported by the data presented?

Yes

-Are the limitations of analysis clearly described?

Yes

-Do the authors discuss how these data can be helpful to advance our understanding of the topic under study?

Yes

-Is public health relevance addressed?

Yes

**Response 22:**

We appreciate the kind evaluations.

**Summary and General Comments**

Reviewer #1: This paper lacks novelty. there is no new in the results of the study and it is not contribute to the readers rather than the scientific community. I ask where is the new findings or contribution in the field?. All methods are routinely used in the similar studies, also the results obtained have no impact in the scientific community

**Response 23:**

We accepted the summary sincerely. We removed the claim about the novelty of methodology from the draft and emphasized the outcome that detected various mycetoma pathogens at the species level from the soil in an endemic area. Hopefully, the results obtained will contribute to our scientific knowledge of microbiology in an arid environment.

Reviewer #2: I have reviewed the manuscript entitled “A novel method of an environmental study of multiple mycetoma pathogens: Matabarcoding análisis for soil DNA of the endemic área in Sudan”.

The authors use a relatively novel technique and this is remarkable and valuable. However, the wording and clarity must be improved especially in the methodology. Unfortunately authors did not recover M. mycetomatis DNA, as the main eumycetoma agent in Sudan. If the authors improve the writing and clarity of the English (the discussion is fine), the document is salvageable.

**Response 24:**

We are grateful for the supportive comments. Through the secondanalysis with the different gene regions, we successfully detected the primary pathogen. Moreover, the draft of this version was modified by an English editing service.

Reviewer #3: The authors performed an analysis of soil samples in several locations in Sudan with the objective of detecting eumycetoma causing agents. The study is well performed and the methodology thoroughly detailed. The findings are interesting and relevant due to the scarcity of environmental studies of these fungi. Please include in the methodology section the month and usual weather when the soil samples were retrieved as these may influence future studies that aim to isolate or detect these microorganisms. Please include in the discussion section the reasons you believe of M. mycetomatis was not found.

**Response 25:**

We appreciate the summary and suggestion about the weather. We added the information of average temperature and humidity of sampling month as retrieved from a reliable data source.
